# Immunotherapies for pediatric cancer: current landscape and future perspectives

**DOI:** 10.1007/s10555-019-09819-z

**Published:** 2019-12-11

**Authors:** Brian Hutzen, Siddhi Nath Paudel, Meisam Naeimi Kararoudi, Kevin A. Cassady, Dean A. Lee, Timothy P. Cripe

**Affiliations:** 1 The Abigail Wexner Research Institute at Nationwide Children’s Hospital Center for Childhood Cancer and Blood Disorders, 575 Children’s Crossroad, Columbus, OH 43215 USA; 2 Graduate Program in Molecular, Cellular and Developmental Biology, The Ohio State University, Columbus, OH USA; 3 Division of Hematology/Oncology/BMT, Department of Pediatrics, Nationwide Children’s Hospital, Columbus, OH USA; 4 Ohio State University Wexner College of Medicine, Columbus, OH USA

**Keywords:** Pediatric cancer, Immune checkpoint inhibitors, Bispecific antibodies, Cell-based therapies, Tumor microenvironment

## Abstract

The advent of immunotherapy has revolutionized how we manage and treat cancer. While the majority of immunotherapy-related studies performed to date have focused on adult malignancies, a handful of these therapies have also recently found success within the pediatric space. In this review, we examine the immunotherapeutic agents that have achieved the approval of the US Food and Drug Administration for treating childhood cancers, highlighting their development, mechanisms of action, and the lessons learned from the seminal clinical trials that ultimately led to their approval. We also shine a spotlight on several emerging immunotherapeutic modalities that we believe are poised to have a positive impact on the treatment of pediatric malignancies in the near future.

## Introduction

While advancements in chemo- and radiotherapy have had a remarkable impact on the overall survival of children afflicted with cancer over the past few decades, pediatric malignancies continue to be a leading cause of death by disease in people younger than 20 years of age. In addition, many patients that survive into adolescence and beyond often do so with a host of debilitating treatment-related side effects that can permanently impact their quality of life. Immunotherapies are an emerging form of treatment that are designed to help the patient’s immune system eradicate cancerous cells while mitigating many of the unfortunate sequelae associated with conventional therapies. Numerous forms of immunotherapy have shown promising results in adult malignancies, paving the way for their implementation against various types of childhood cancer. In this review, we highlight those immunotherapies that have attained approval of the US Food and Drug Administration for treatment of pediatric malignancies, providing insight on their development, mechanisms of action, and their use in the clinic. We also examine some of the new immunotherapeutic approaches on the horizon, focusing on their development and the challenges and opportunities that lie ahead as they make their way into clinical application.

## Immunotherapies approved by the FDA for the treatment of childhood cancer

### Blinatumomab (Amgen)

Blinatumomab (trade name Blincyto®) is the first-in-class member of a group of novel-targeted antibodies known collectively as bispecific antibodies (BsAbs). These molecules are typically constructed of two single-chain variable fragments (scFv) joined by a flexible linker. While there is room for variation, most BsAbs described to date share a similar design in that one scFv is specific for a validated tumor antigen, whereas the other is targeted to the CD3 subunit of the T cell receptor [1]. By physically bridging T cells to tumor cells, BsAbs facilitate the formation of an immunological synapse inducing T cell degranulation, culminating in the death of the conjoined tumor cell [2]. In the case of blinatumomab, the tumor-targeting arm of the BsAb is an scFv specific for the B lymphocyte antigen CD19, which is expressed on nearly all B-lineage lymphoblastic leukemias and lymphomas [3].

Blinatumomab was first introduced in 2000 by Löffler and colleagues, who showed that this construct promoted significant cytotoxicity in CD19^+^ lymphoma cells co-cultured with unstimulated T cells at concentrations of 10–100 pg/mL and effector to target cell ratios as low as 2:1 [4]. Results from the first clinical trial to feature blinatumomab, a study on 38 adult patients with relapsed or refractory non-Hodgkin lymphoma, were published in 2008 [5]. Partial and complete tumor regressions were observed at dose levels as low as 0.015 mg/m^2^/d given over 4 to 6 weeks, and all seven patients enrolled in the 0.06-mg dose level arm of the study showed objective responses. A phase II trial for acute lymphoblastic leukemia (ALL) in adults was conducted shortly thereafter, where it was concluded that blinatumomab treatment was efficacious and generally well tolerated [6, 7].

The first use of blinatumomab in pediatric patients was made possible under a compassionate use approval for treatment of post-transplant relapsed B cell precursor ALL (B cell ALL) [8]. Three patients, having failed to achieve durable responses following allogenic bone marrow transplantation and multiple cycles of chemotherapy, were given blinatumomab as a continuous intravenous infusion (0.015 mg/m^2^/d) for several weeks. Although each patient started treatment with different levels of leukemia load, blinatumomab administration induced an expansion of donor-derived T lymphocytes and lowered their level of leukemic blast cells below the threshold of minimal residual disease; one of these patients was still in complete remission as of 2018 [9]. Compelling results from an additional cohort of six pediatric patients spurred the initiation of an open-label phase I/II study for use of blinatumomab to treat chemotherapy-refractory B cell ALL in 2011 [10, 11]. Data collected from 49 patients in the phase I arm of the study were used to determine an optimal dosing regimen. This regimen consisted of 6-week treatment cycles starting with a 7-day period of low dose (0.005 mg/m^2^/d) blinatumomab to reduce the risk of immunological toxicities and increasing it to a higher 0.015 mg/m^2^/d dose thereafter. The most common adverse events observed were pyrexia (80%), anemia (41%), nausea (33%), and headache (30%); the majority of which were reported within the first few days of induction. Administration of the drug was also noted to be somewhat cumbersome, as it requires continuous infusion due to its short (~ 2 h) half-life. Blinatumomab treatment was temporarily halted in 10 patients and discontinued in 4 others because of the severity of their adverse events, including two incidents of cytokine release syndrome. Of the 70 patients treated in the phase II arm of the study, 39% achieved complete remission (defined as no evidence of blasts or extramedullary disease) within two treatment cycles; more than half of these responding patients would eventually achieve a complete minimal residual disease response [10].

In the wake of these overall promising results, blinatumomab received FDA approval for use in pediatric patients with Philadelphia chromosome-negative relapsed/refractory B cell ALL in 2016. This approval was expanded the following year to include adults and pediatric patients with relapsed or refractory B cell ALL and expanded again in 2018 to treat adults and children with B cell ALL who are in remission but still have minimal residual disease [12]. Additionally, Amgen recently announced that enrollment in two of their ongoing phase III trials comparing the efficacy, safety, and tolerability of blinatumomab to conventional chemotherapy in pediatric B cell ALL patients (NCT02393859 and COG-AALL1331) had been terminated early due to the lower toxicity, better minimal disease clearance, and improved disease-free and overall survival observed in the blinatumomab arms of each study [13].

### Dinutuximab (United Therapeutics)

Dinutuximab (trade name Unituxin®) is a chimeric human-mouse antibody specific for disialoganglioside (GD2), a glycolipid antigen that is highly expressed on the surface of neuroblastoma and a variety of other embryonal tumors including rhabdomyosarcoma, Ewing sarcoma, retinoblastoma, osteosarcoma, and some pediatric brain tumors [14, 15]. Originally known as “ch14.18,” dinutuximab was developed in the late 1980s alongside several other clinical-grade anti-GD2 antibodies as a treatment for neuroblastoma [16]. These antibodies elicit their antitumor effects by binding GD2 on the tumor cell surface, allowing their respective Fc portions to engage receptors on monocytes, macrophages, neutrophils, and natural killer cells. This engagement subsequently triggers the death of the tumor cell by antibody-dependent cell-mediated cytotoxicity (ADCC) and/or complement-dependent cytotoxicity [17–19].

Dinutuximab is the combination of the variable regions of the murine IgG3 anti-GD2 monoclonal antibody 14.G2a and the constant regions of human IgG1 [20]. This design choice was taken as a precautionary measure to diminish the chances of the patient developing a human anti-mouse antibody (HAMA) response following administration, which can negatively impact antitumor efficacy. Due to a combination of promising preclinical data and early clinical results with the 14.G2a antibody, a phase I clinical trial to determine the toxicity and maximum tolerated dose (MTD) of dinutuximab in stage 4 neuroblastoma patients was initiated in the early 1990s [21]. Nine pediatric patients were treated with 19 courses of dinutuximab at dose levels of 30, 40, or 50 mg/m^2^/d and 5 days per course. An MTD of 50 mg/m^2^ per injection was established with noted side effects of urticaria, pruritus, and pain, the latter of which is now believed to be the result of complement activation at GD2-expressing nerve fibers (though it predominantly occurs only during infusion when there are peak serum levels) [22]. Over the course of the trial, four patients achieved a complete or partial remission, two others displayed a minor response or stable disease, and three patients showed tumor progression. Importantly, and in direct contrast to earlier studies with the 14.G2a antibody, no patient developed a HAMA response [21].

Following these data, a larger study was initiated with 164 patients who were treated under the German neuroblastoma protocol NB97. Patients given at least one cycle of dinutuximab after intensive chemotherapy exhibited a better overall survival rate compared with patients who did not receive consolidation treatment [23]. Subsequent clinical trials, where dinutuximab was combined with immune adjuvant granulocyte-macrophage colony stimulating factor (GM-CSF) to enhance ADCC, also showed promising efficacy and tolerability [19, 24]. These findings precipitated a phase I study by the Children’s Oncology Group in 1997, where the combination of dinutuximab with GM-CSF and interleukin-2 (IL-2) was investigated in 25 pediatric patients following high dose chemotherapy with autologous bone marrow rescue [25]. This study established an MTD of 25 mg/m^2^/d dinutuximab given concurrently with alternating courses of 4.5 × 10^6^ U/m^2^/d of IL-2 or 0.25 mg/m^2^ GM-CSF for 6 total courses at 28-day intervals. Although two patients experienced dose-limiting toxicities on this regimen, no deaths were attributable to toxicity of the therapy and no patient developed a HAMA response.

These findings paved the way for the Children’s Oncology Group ANBL0032 randomized phase III study in 2001, which examined the benefits of adding dinutuximab with GM-CSF and IL-2 to standard isotretinoin therapy in high-risk neuroblastoma patients [26]. The study, which randomly assigned 226 patients into immunotherapy and standard therapy groups, was stopped prematurely in 2009 due to the superiority of the dinutuximab-treated group with regard to event-free survival (66% *versus* 46% at 2 years) and overall survival (86% *versus* 75% at 2 years). Treatment-related side effects were most common during the first cycle of treatment and generally consistent with expectations of dinutuximab and IL-2 therapy, including pain (52% of patients), hypotension (18% of patients), capillary leak syndrome (23% of patients), and hypersensitivity reactions (25% of patients). In light of this success, the FDA-approved dinutuximab combination therapy in 2015 for high-risk neuroblastoma patients who achieve at least a partial response to frontline multimodal therapy [27]. A variant of dinutuximab known as dinutuximab-beta (trade name Qarziba®), which is produced in a different cell line but otherwise displays comparable activities, was similarly approved by the European Commission for high-risk neuroblastoma in 2017 following positive results in another series of clinical studies [28, 29]. Efforts to further improve the efficacy of dinutuximab and lower its incidence of side effects (e.g., by further humanization of the antibody or altering its rate of administration) are currently under investigation [19].

### Pembrolizumab (Merck) and ipilimumab (Bristol-Myers Squibb)

Pembrolizumab (trade name Keytruda®) and ipilimumab (trade name Yervoy®) are two of the most prominent members of a class of immunotherapeutics collectively known as immune modulators or immune checkpoint inhibitors. While the targets of these two antibodies are distinct, both function by impeding inhibitory signals of T cell activation which in turn allows these cells to better mount an effective antitumor response [30–32].

Pembrolizumab is a humanized IgG4 monoclonal antibody specific for programmed cell death protein 1 (PD-1), a cell surface receptor expressed on activated T and B lymphocytes [33]. PD-1 negatively regulates T cell activation through engagement of its ligands, PD-L1 and PD-L2, which are widely expressed in non-lymphoid tissues and further upregulated in response to inflammatory cytokines [34]. Engagement of PD-L1 or PD-L2 by PD-1 results in the attenuation of T cell activity through negative regulation of proximal signaling elements of the T cell receptor [35]. Although the feedback loop enabled by the PD-1 signaling axis is essential for maintaining peripheral tolerance and preventing autoimmunity, malignant tumors can also co-opt these processes by upregulating PD-L1 and/or PD-L2 to shield themselves from immune destruction [32, 36]. While the exact mechanism(s) of how pembrolizumab and similar checkpoint inhibitors achieve their antitumor activity remain to be fully elucidated, PD-1 blockade has been shown to “reinvigorate” and expand exhausted T cells in the tumor microenvironment, thereby helping promote tumor rejection [37].

Originally known as MK-3475 (and later designated “lambrolizumab”), pembrolizumab was developed in 2006 and later acquired by Merck in 2009. A first-in-human phase I clinical trial involving adult patients with advanced solid tumors was initiated shortly thereafter. Results from this study were published in 2015 and showed clinical responses at all pembrolizumab dose levels tested (1, 3, or 10 mg/kg every 2 weeks) without reaching dose-limiting toxicities [38]. Subsequent clinical trials were started in earnest (see reference [39] for a more comprehensive review), and positive results from these studies eventually culminated in the FDA approval of pembrolizumab for the treatment of more than 20 indications including melanoma in 2014 [40], non-small cell lung cancer in 2015 [41], head and neck squamous cell carcinoma in 2016 [42], Hodgkin lymphoma in 2017 [43], gastric and gastroesophageal carcinoma in 2018 [44], renal cell carcinoma [45], and certain forms of endometrial cancer in 2019 [46].

Despite these successes, relatively few studies examining the use of pembrolizumab to treat pediatric malignancies have been conducted to date. In a 2014 phase I study of a PD-1 targeted antibody in children, the Sarcoma Alliance for Research through Collaboration investigated the use of single therapy pembrolizumab in pediatric patients with advanced soft tissue or bone sarcomas [47]. A total of 84 patients received 200 mg of pembrolizumab intravenously every 3 weeks, and while the drug was well tolerated, an objective response (as assessed by the investigators) was only achieved in 18% of soft tissue sarcoma patients and 5% of bone sarcoma patients. Pembrolizumab would eventually be approved by the FDA in 2017 for adults and children with refractory Hodgkin lymphoma or patients who relapsed after three or more prior treatments. This decision was based on efficacy data obtained from the KEYNOTE-087 trial, where 210 adult Hodgkin lymphoma patients treated with pembrolizumab showed an overall response rate of 69%, with 22% achieving a complete remission; efficacy for the pediatric population was extrapolated from these results as drug tolerability at the adult level had previously been established [48]. As of this writing, Hodgkin lymphoma remains the only pediatric indication for pembrolizumab therapy.

Ipilimumab is a fully humanized monoclonal IgG1 antibody that targets cytotoxic T lymphocyte antigen 4 (CTLA-4). Like PD-1, CTLA-4 is intrinsically linked with T cell activation. Its expression is upregulated immediately after T cell receptor engagement where it acts to dampen T cell activation by competing with the CD28 receptor for access to the costimulatory molecules B7-1 (CD80) and B7-2 (CD86) [32, 49–51]. Ipilimumab sterically blocks CTLA-4 and prevents it from making these interactions. This in turn has been shown to enhance T cell activation, increase neoantigen-specific CD8 T cells in the tumor microenvironment, and deplete immunosuppressive T regulatory cells [52, 53].

Originally known as MDX-010, ipilimumab was developed in the early 2000s by the biopharmaceutical company Medarex and later acquired by Bristol-Myers Squibb in 2009 [54, 55]. Following encouraging preclinical data, results from the first human phase I clinical trial to feature ipilimumab were published in 2007 [56]. The objectives of this study were to determine the safety of a single 3 mg/kg dose of ipilimumab (previously found safe in macaque toxicity studies) in 14 patients with hormone-refractory prostate cancer and to assess whether treatment modulated T cell activation or expression of prostate-specific antigen. Ipilimumab treatment was generally well tolerated with only one patient developing clinical autoimmunity, which manifested as a grade 3 rash/pruritus that cleared with corticosteroid treatment. Although there was no change in patient lymphocyte populations over time save for a slight uptick in CD4 and CD8 T cells expressing the T cell activation marker HLA-DR, two patients showed a ≥ 50% decrease in levels of prostate-specific antigen. Pharmacokinetic analyses also revealed a circulating half-life of 12.5 days for ipilimumab, suggesting a 3- or 4-week dosing regimen could be feasible for subsequent clinical studies.

Numerous phase I/II studies evaluating ipilimumab in various cancers have been conducted in the years since [57], but its greatest success to date has been in the management of malignant melanoma for which it received FDA approval in 2011 [58, 59]. Results from the ipilimumab registration trial were reported in the New England Journal of Medicine in 2010 [60]. In this phase III study, a total of 676 advanced melanoma patients were randomized to receive treatment consisting of ipilimumab (3 mg/kg every 3 weeks, 4 doses total), a glycoprotein 100 (gp100) peptide vaccine, or the combination thereof. Patients who received ipilimumab, either alone or as combination therapy, exhibited significantly improved overall survival compared with patients treated with gp100 (~ 10 months *vs.* 6.4 months), with 1-year overall survival rates of 45.6% for ipilimumab alone, 25.3% for gp100 alone, and 43.6% for their combination. Adverse events were reported in ~ 60% of ipilimumab-treated patients and 32% of gp100-treated patients. These events predominately affected the skin and gastrointestinal tract and occurred most frequently at the time of induction, while most of these events were manageable with corticosteroid treatment, 10–15% were classified as grade 3 or 4 and a total of 14 deaths (2.1%) were ultimately attributed to the study drugs.

In 2017, the FDA expanded the approval of ipilimumab to treat unresectable or metastatic melanoma in pediatric patients ≥12 years of age [61]. This approval was granted in consideration of data from two early phase clinical studies. The first, a phase I dose-finding study in 33 patients with advanced solid tumors, established that a 3 mg/kg dose of ipilimumab was generally well tolerated in pediatric patients, who exhibited drug-related toxicities and pharmacokinetic profiles similar to those previously observed in adult studies [62]. The second study investigated the use of 3 mg/kg (*n* = 4) or 10 mg/kg (*n* = 8) dosing of ipilimumab in 12 adolescents with stage 3 or 4 malignant melanoma [63]. Although this phase II trial was stopped early due to the slow accrual of patients, the available data were promising: after 1 year, 75% of the patients treated at 3 mg/kg and 62.5% of patients treated at 10 mg/kg were still alive.

Although immune modulators continue to break new ground in the treatment of adult malignancies, successes in the pediatric setting have generally been elusive. As mentioned above, checkpoint inhibitors bolster antitumor immunity by lifting some of the restraints the tumor microenvironment imposes on T cells. The strength of the tumor-specific T cell response is in turn highly influenced by the mutational landscape or neoantigen load of the tumor itself [64]. One of the hallmarks of most pediatric cancers, however, is their relative lack of mutations (a rare exception are cancers arising from constitutional mismatch repair deficiencies, which do respond to checkpoint inhibition therapy) [65]. Preclinical data also suggest that tumors can develop compensatory mechanisms to circumvent immunotherapy if treatment is limited to a single approach [66, 67]. Combining checkpoint inhibitors is one strategy that may help overcome these obstacles [68]. In support of this concept, multiple phase I/II trials investigating combined PD-1/CTLA-4 signaling blockade in pediatric malignancies are presently underway [69–71].

### Tisagenlecleucel (Novartis)

Tisagenlecleucel (trade name Kymriah®) is a chimeric antigen receptor (CAR)-T cell therapy for relapsed and/or refractory B cell malignancies. Using the patient’s own T cells as a starting point, CAR-T cells are genetically modified to combine the extracellular antigen recognition domain of an antibody with the intracellular signaling domain of the T cell receptor (TCR). In the case of tisagenlecleucel, this antigen recognition domain is an scFv derived from the mouse monoclonal antibody FMC63, which specifically binds human CD19 in its native conformation [72]. This domain is in turn linked to the TCR domain by transmembrane and spacer domains, which give its added flexibility to optimally engage CD19-expressing cancer cells. As a “second-generation” CAR-T therapy, tisagenlecleucel has also been designed to express a 4-1BB (CD137) costimulatory domain to promote T cell proliferation and persistence in the patient following administration [73]. CAR-T cells are an attractive form of immunotherapy because they can engage tumor cells independently of antigen presentation by the major histocompatibility complex (MHC), which is often downregulated or absent in many cancers as a defense against immune-mediated destruction [74].

Formerly known as CTL019, tisagenlecleucel was developed in the early 2010s by researchers at the Children’s Hospital of Philadelphia and later in collaboration with the pharmaceutical company Novartis following promising results from early CD19-targeted CAR-T pilot studies [73, 75–78]. Tisagenlecleucel was first utilized in a small phase I/IIA study of two pediatric patients with relapsed and refractory pre-B cell ALL in 2012 [79]. These patients received transfusions of tisagenlecleucel at a dose of 1.4 × 10^6^–1.2 × 10^7^ cells per kilogram of body weight. Levels of circulating lymphocytes were found to have expanded dramatically in each patient, with the levels of CD19-targeted CAR-T cells increasing ≥ 1000 times their initial engraftment and persisting for several months. Each patient exhibited acute toxic side effects such as cytokine release syndrome and B cell aplasia, but these were reversible (cytokine release syndrome) or tolerable (aplasia) and did not negatively impact expansion of tisagenlecleucel or diminish its anti-leukemic effects. Complete remissions were achieved by both patients; although one patient eventually relapsed with CD19^−^ blasts, the other has remained cancer-free for 7 years as of May 2019 in what is considered one of the most resounding success stories of immunotherapy to date [80].

Two subsequent tisagenlecleucel studies were initiated later in 2012. The first was a phase I trial in 30 patients (25 between the ages of 5 and 22) with relapsed B cell ALL [81]. These patients were transfused with 7.6 × 10^5^ to 2.06 × 10^7^ tisagenlecleucel cells per kilogram of body weight and monitored for a response, including CAR-T cell expansion and persistence and the development of any toxicities. Complete remissions were achieved in 90% of the patients, including two patients who were previously refractory to blinatumomab therapy. Tisagenlecleucel expansion was documented in all responding patients, as was the development of manageable symptoms of cytokine release syndrome. Sustained remission was achieved with 6-month event-free and overall survival rates of 67% and 78%, respectively. The other trial, conducted in 21 pediatric and young adult ALL patients, established an MTD 1 × 10^6^ tisagenlecleucel cells per kilogram of body weight [82]. Complete remissions were observed in 70% of these patients, and all associated toxicities, including 3 cases of grade 4 cytokine release syndrome, were fully reversible.

Promising results from these studies paved the way for the pivotal phase II ELIANA study (NCT02435849) in 2015, which evaluated the safety and efficacy of tisagenlecleucel in 75 pediatric and young adult B cell ALL patients [83, 84]. The primary endpoint of this study was an overall remission rate ≥ 20%, which was defined as complete remission with or without complete hematologic recovery within 3 months of follow-up; an 81% overall remission rate was observed within this timeframe, dropping to a still impressive 73% at 6 months and 50% at 12 months. Although tisagenlecleucel was generally well tolerated, every patient had at least one adverse event over the course of the study. The most common side effects included cytokine release syndrome (77%), pyrexia (40%), neurologic events (40%), decreased appetite (39%), febrile neutropenia (36%), and headache (36%). Every patient showing a response to treatment also exhibited B cell aplasia, which was managed with immunoglobulin replacement therapy (and is notably required monthly as long as the CAR-T cells remain active) [83]. Based on these data, tisagenlecleucel was approved by the FDA in 2017 for the treatment of refractory/relapsed B cell ALL in patients ≤ 25 years old [85], and multiple clinical trials examining its effectiveness against other B cell malignancies are actively being pursued [86–88].

## Emerging immunotherapies for the treatment of childhood cancer

Immunotherapy is an incredibly diverse and rapidly progressing field. While the majority of current studies are decidedly focused on adult disease, considerable efforts are being made to bring these therapies into the pediatric space; currently, more than three-dozen early phase clinical trials evaluating immunotherapies for childhood cancers are active and/or recruiting (Table 1). In the sections below, we highlight a few of the emerging forms of immunotherapy that, while currently not FDA-approved, are nonetheless poised to have a large impact in the treatment of pediatric malignancies in the near future.

**Table 1:** Pediatric immunotherapy clinical trials that are currently active and/or recruiting

Study name	Phase	Disease(s)	Agent(s)	Modality	Identifier	Reference
Pediatric precision laboratory advanced neuroblastoma therapy	II	Neuroblastoma	Ceritinib	Small molecule inhibitors	NCT02559778	
			Dasatinib			
			Sorafenib			
			Vorinostat			
			α-difluoromethylornithine			
Durvalumab and tremelimumab for pediatric malignancies	I/II	Advanced solid tumors and hematological malignancies	Durvalumab	Immune modulators (PD-1 and CTLA-4)	NCT03837899	[71]
			Tremelimumab			
			Combination therapy			
Glypican 3-specific chimeric antigen receptor expressed in T cells for patients with pediatric solid tumors (GAP)	I	Pediatric liver cancer	GAP-specific T cells	CAR-T cells in combination with chemotherapy	NCT02932956	
			Cytoxan			
			Fludara			
EGFR806-specific CAR T cell locoregional immunotherapy for EGFR-positive recurrent or refractory pediatric CNS tumors	I	Recurrent or refractory EGFR+ tumors of the CNS	EGFR806-specific T cells	CAR-T cells	NCT03638167	
Famitinib plus camrelizumab *versus* famitinib alone in advanced osteosarcoma	I/II	High-grade osteosarcoma	Famitinib combined with camrelizumab	Receptor tyrosine kinase inhibitor and immune modulator (PD-1)	NCT04044378	[89]
HER2-specific CAR T Cell locoregional immunotherapy for HER2-positive recurrent/refractory pediatric CNS tumors	I	Recurrent or refractory HER2+ tumors of the CNS	HER2-specific T cells	CAR-T cells	NCT03500991	
Engineered neuroblastoma cellular immunotherapy (ENCIT)-01	I	Neuroblastoma and ganglioneuroblastoma	CD171-specific T cells expressing EGFRt (multiple formats)	CAR-T cells	NCT02311621	
An investigational immuno-therapy study of nivolumab monotherapy and nivolumab in combination with ipilimumab in pediatric patients with high grade primary CNS malignancies	II	High-grade primary tumors of the CNS	Nivolumab	Immune modulators (PD-1 and CTLA-4)	NCT03130959	[70]
			Ipilimumab			
EGFR806 CAR T cell immunotherapy for recurrent/refractory solid tumors in children and young adults	I	Pediatric solid tumors	4-1BBζ EGFR806-EGFRt and 4-1BBζ CD19-Her2tG T cells	CAR-T cells	NCT03618381	
Adoptive cellular therapy in pediatric patients with high-grade gliomas	I	High-grade glioma	Total tumor RNA DC vaccine	Cancer vaccine and stem cell transfer	NCT03334305	[90]
			Total tumor RNA with ALT			
			Autologous HSCs			
			Td vaccine			
CD19/CD22 chimeric antigen receptor (CAR) T cells in children and young adults with recurrent or refractory CD19/CD22-expressing B cell malignancies	I	B cell leukemia/lymphoma	CD19/CD22-specific T cells	CAR-T cells in combination with chemotherapy	NCT03448393	
			Fludarabine			
			Cyclophosphamide			
Allogeneic tumor cell vaccination with oral metronomic cytoxan in patients with high-risk neuroblastoma	I/II	Neuroblastoma	SKNLP cells; SJNB cells engineered to secrete interleukin-2 or lymphotactin	Cancer vaccine in combination with chemotherapy	NCT01192555	[91]
			Cytoxan			
Study of the IDO pathway inhibitor, indoximod, and temozolomide for pediatric patients with progressive primary malignant brain tumors	I	High-grade primary tumors of the CNS	Indoximod	Tumor microenvironment modulation combined with chemotherapy/radiotherapy	NCT02502708	
			Temozolomide			
			Cyclophosphamide			
			Etoposide			
			Conformal radiation			
Phase II trial of nivolumab for pediatric and adult relapsing/refractory ALK+ anaplastic large cell lymphoma, for evaluation of response in patients with progressive disease (cohort 1) or as consolidative immunotherapy in patients in complete remission after relapse (cohort 2)	II	Recurrent or refractory ALK+ anaplastic large cell lymphoma	Nivolumab	Immune modulator (PD-1)	NCT03703050	
Immunotherapy (nivolumab or brentuximab vedotin) plus combination chemotherapy in treating patients with newly diagnosed stage III–IV classic Hodgkin lymphoma	III	Hodgkin lymphoma (various subtypes)	Nivolumab	Immune modulator (PD-1) Targeted antibody (CD30) Chemotherapy	NCT03907488	
				Radiotherapy		
			Brentuximab vedotin			
			Filgrastim (G-CSF)			
			Pegfilgrastim (G-CSF)			
			Doxorubicin			
			Dacarbazine			
			Vinblastine			
			Radiation			
Research study utilizing expanded multi-antigen specific lymphocytes for the treatment of solid tumors	I	Pediatric solid tumors	Multi-antigen specific T cells	Adoptive cell transfer	NCT02789228	
Anti-CD22 chimeric receptor T cells in pediatric and young adults with recurrent or refractory CD22-expressing B cell malignancies	I	Follicular lymphoma, ALL, non-Hodgkin’s lymphoma, large cell lymphoma	CD22-specific T cells	CAR-T cells	NCT02315612	
Brain stem gliomas treated with adoptive cellular therapy during focal radiotherapy recovery alone or with dose- intensified temozolomide	I	Diffuse intrinsic pontine glioma	Total tumor RNA DC vaccine	Cancer vaccine, chemotherapy, and stem cell transfer	NCT03396575	[92]
			Total tumor RNA with ALT			
			Cyclophosphamide			
			Fludarabine			
			Temozolomide			
			Td vaccine			
			Autologous HSCs			
Phase 1b study PVSRIPO for recurrent malignant glioma in children	I	High-grade glioma	PVSRIPO (polio/rhinovirus recombinant)	Oncolytic virus	NCT03043391	[93]
HSV G207 in children with recurrent or refractory cerebellar brain tumors	I	Pediatric brain tumors	G207 (Herpes virus recombinant)	Oncolytic virus	NCT03911388	[94]
HSV G207 alone or with a single radiation dose in children with progressive or recurrent supratentorial brain tumors	I	Pediatric brain tumors	G207	Oncolytic virus with radiotherapy	NCT02457845	
			Radiation			
Oncolytic adenovirus, DNX-2401, for naive diffuse intrinsic pontine gliomas	I	Diffuse intrinsic pontine glioma	DNX-2401 (adenovirus recombinant)	Oncolytic virus	NCT03178032	[95]
Modified measles virus (MV-NIS) for children and young adults with recurrent medulloblastoma or recurrent ATRT	I	Medulloblastoma, atypical teratoid/rhabdoid tumor	MV-NIS (measles virus recombinant)	Oncolytic virus	NCT02962167	
Wild-type reovirus in combination with sargramostim in treating younger patients with high-grade relapsed or refractory brain tumors	I	Pediatric brain tumors	Reovirus	Oncolytic virus with immunostimulatory cytokine	NCT02444546	
			Sargramostim (GM-CSF)			
Vigil + irinotecan and temozolomide in Ewing’s sarcoma (VITA)	III	Ewing sarcoma family of tumors	Vigil	Autologous whole cell cancer vaccine with chemotherapy	NCT03495921	[96]
			Irinotecan			
			Temozolomide			
Haploidentical stem cell transplantation in neuroblastoma	I	Neuroblastoma	Haploidentical stem cells, mesenchymal stem cells fludarabine, thiotepa rituximab, I131-MIBG	Stem cell transfer with chemotherapy and targeted radiotherapy	NCT00790413	
Durvalumab in pediatric and adolescent patients	I	Pediatric solid tumors	Durvalumab	Immune modulator (PD-1)	NCT02793466	
		Lymphoma				
		CNS tumors				
T-lymphocytes genetically targeted to the B-cell specific antigen CD19 in pediatric and young adult patients with relapsed B-cell acute lymphoblastic leukemia	I	Relapsed B cell ALL	CD19-specific T cells	CAR-T cells combined with chemotherapy	NCT01860937	
			Cyclophosphamide			
A phase II trial of avelumab in patients with recurrent or progressive osteosarcoma	II	Osteosarcoma	Avelumab	Immune modulator (PD-L1)	NCT03006848	
A phase I study of AdV-tk + prodrug therapy in combination with radiation therapy for pediatric brain tumors	I	Malignant glioma recurrent ependymoma	AdV-tk (adenovirus)	Oncolytic virus	NCT00634231	[97]
			Valacyclovir	Anti-herpetic prodrug Radiotherapy		
			Radiation			
A two-part phase IIb trial of vigil in Ewing’s sarcoma	II	Ewing sarcoma	Vigil	Autologous whole cell cancer vaccine with chemotherapy	NCT02511132	[98]
			Irinotecan			
			Temozolomide			
PEP-CMV in recurrent medulloblastoma/malignant glioma	I	Malignant glioma medulloblastoma	PEP-CMV (peptide vaccine derived from Cytomegalovirus)	Cancer vaccine	NCT03299309	[99]
A pediatric and young adult trial of genetically modified T cells directed against CD19 for relapsed/refractory CD19+ leukemia	I/II	Leukemia	CD19-specific T cells expressing EGFRt	CAR-T cells	NCT02028455	
Treatment protocol for children and adolescents with acute lymphoblastic leukemia - AIEOP-BFM ALL 2017	III	B cell ALL	Blinatumomab	Bispecific T cell engager with chemotherapy	NCT03643276	
		T cell ALL	SoC chemotherapy drugs			
A phase 1 study of CD22-CAR T cell immunotherapy for CD22+ leukemia and lymphoma	I	Leukemia	CD22-specific T cells expressing EGFRt	CAR-T cells	NCT03244306	
		Lymphoma				
Pilot study of T-APCs following CAR T cell immunotherapy for CD19+ leukemia	I	Leukemia	T cell antigen presenting cells expressing truncated CD19	Autologous T cell transfer	NCT03186118	
Pembro + blina combination in pediatric and young adult patients with relapsed/refractory acute leukemia or lymphoma	I	B cell leukemia	Pembrolizumab	Immune modulator (PD-1) combined with bispecific T cell engager	NCT03605589	
		B cell lymphoma	Blinatumomab			
A feasibility and safety study of dual specificity CD19 and CD22 CAR-T cell immunotherapy for CD19+CD22+ leukemia	I	Leukemia	CD19/CD22-specific T cells	CAR-T cells	NCT03330691	
		Lymphoma				
Her2 chimeric antigen receptor expressing T cells in advanced sarcoma	I	Pediatric sarcomas	HER2-specific T cells	CAR-T cells combined with chemotherapy	NCT00902044	
			Fludarabine			
			Cyclophosphamide			
Tabelecleucel in combination with pembrolizumab in subjects with epstein-barr virus-associated nasopharyngeal carcinoma	I/II	Recurrent/metastatic EBV-associated nasopharyngeal carcinoma	Tabelecleucel	Autologous T cell transfer combined with immune modulator (PD-1)	NCT03769467	
			Pembrolizumab			
H3.3K27M peptide vaccine for children with newly diagnosed DIPG and other gliomas	I	Diffuse intrinsic pontine glioma	K27M peptide	Cancer vaccine	NCT02960230	[100]
		Malignant glioma				
Therapy for pediatric relapsed or refractory precursor B-cell acute lymphoblastic leukemia and lymphoma	II	Recurrent B cell ALL and B-lymphoblastic lymphoma	Rituximab, NK cell infusion, autologous HSC transplant, and SoC chemotherapy drugs	Targeted antibody (CD20), NK cell and stem cell transfer combined with chemotherapy	NCT01700946	

This list was compiled from https://clinicaltrials.gov on September 30, 2019, using the search terms “pediatric cancer,” “oncolytic virus,” and “immunotherapy” filtered for active and/or recruiting trials

### Oncolytic virotherapy

Oncolytic viruses (OVs) are either non-pathogenic wild type viruses or genetically modified attenuated pathogenic viruses that selectively kill cancer cells directly through lysis or indirectly by stimulating an antitumor immune response. This selectivity for transformed cells can either be intrinsic or artificially engineered depending upon the virus. Virus replication is typically restricted to cancer cells due to genetic defects in their anti-viral response pathways that, while advantageous for cancer proliferation, also benefit production of nascent virions. As a further safety measure, genes necessary for virus replication in normal cells can be deleted or expressed under the control of a promoter specific to the cancer. Although both lytic potential and antitumor immunity are equally enticing for therapy, the field has focused primarily on the immune response arm of OVs as it has a greater potential benefit in most contexts [101, 102].

The only FDA-approved oncolytic virus to date is talimogene laherparepvec (T-VEC), an attenuated herpes simplex virus type-1 (HSV-1) that expresses the transgene for human GM-CSF [103]. Although it is currently only approved for advanced melanoma in adults, a number of similar viruses are being studied in pediatric cancers. These include oncolytic adenovirus, new castle disease virus, polio virus, herpes simplex virus, vesicular stomatitis virus, reovirus, measles virus, and vaccinia virus. Despite having a different genetic landscape from adult cancers, OVs have shown promising preclinical results in pediatric tumor models. Clinical data from two phase I clinical trials of modified herpes simplex virus (NCT00931931) and vaccinia virus (NCT01169584) in extracranial solid tumors have established the safety of these OVs in childhood cancer [104–106]. Virus replication was observed in each of these studies, although no objective responses were reported at the dose levels administered. The vaccinia trial also showed an immunological response after injection. Apart from this, phase I trials for oncolytic adenovirus (NCT00634231 and NCT03178032) and poliovirus (NCT03043391) are currently being tested against pediatric brain tumors [93, 95, 97]. The oncolytic reovirus, Reolysin, has also advanced to phase II clinical testing for bone and soft tissue sarcoma and includes pediatric patients older than 15 years.

Combination strategies have been successfully used to optimize immunotherapy in clinical models in the adult population. Recently, a phase I clinical trial was conducted in adult patients with advanced melanoma by combining T-VEC and anti-PD-1 antibody pembrolizumab [107]. This combination was well tolerated and had no toxic effects. Overall response rate (62%) and complete response rate (33%) were higher than the previously reported result for pembrolizumab alone in a similar setting. This synergy correlated with increased infiltration of CD8 T cells in the responding population, even in patients who had low baseline CD8 T cell density [107]. These studies suggest that immunogenic cell death following OV infection can potentially translate to increased antigen presentation and enhance the effect of other immunotherapies in an adjuvant setting. Although the combination of immune checkpoint blockade with oncolytic virotherapy is yet to be tested in children, an ongoing clinical trial of modified herpes simplex virus G207 against pediatric high grade brain tumors incorporates combination with single low dose of radiation (NCT03911388) [94]. Preclinical and clinical works have expanded the horizon of the application of OVs using multiple strategies. Further work is necessary to realize this potential in its entirety in pediatric population.

### Natural killer cell-based therapies

Natural killer (NK) cells are innate lymphocytes with the ability to eliminate cancers. NK cells, unlike T cells, do not need any prior encounter with cancer cells for their activation. A balance between activating and inhibitory signals initiated from ligand-receptor interaction dictates the NK cells’ function. Activating receptors such as *NKp46*, *NKp44*, *NKp30*, *DNAM1*, and *NKG2D* recognize tumor antigens and activate NK cells. These NK cells secrete cytotoxic granules containing perforin and granzymes that directly kill cancer cells by inducing apoptosis through different mechanisms such as Fas and TRAIL pathways. NK activation in the tumor microenvironment can also be an essential driver for dendritic cells and T cells to be recruited into the tumor site. Moreover, NK cells can be activated *via* ADCC through engaging *CD16 (FcγRIIIA)* with monoclonal antibodies such as daratumumab for targeting multiple myeloma and T-ALL or rituximab for treating B cell non-Hodgkin lymphoma [108, 109].

Additionally, these lymphocytes have anti-viral and anti-graft-*versus*-host disease potential, which makes them an excellent option for adoptive pediatric cancer immunotherapy [110, 111]. In allogeneic stem cell and autologous hematopoietic stem cell transplantation (HSCT), NK cells play an essential role in graft *versus* leukemia. Their cytotoxicity in the setting of leukemia is regulated through several genes such as *KIR*, *NKG2D*, and their ligands. These cells can target several leukemic cell types, spanning acute myeloid leukemia (AML), chronic myeloid leukemia (CML), and chronic lymphocytic leukemia (CLL), with no risk of acute graft *versus* host disease [112–114]. NK cells can be isolated from different sources such as peripheral blood of haploidentical, allogeneic, or autologous donors as well as cord blood and induced pluripotent stem cells (iPSC). To achieve sufficient volumes of NK cells for clinical use, *ex vivo* expansion systems using K562 feeder cells are designed to engage and activate NK cells with membrane bound ligands such as mbIL21-41BBL or mbIL15-41BBL as well as a combination of multiple stimulatory cytokines such as IL-2, IL-12, IL-15, and IL-18 [111]. To avoid using leukemic feeder cells in the culture process, generating small fragments of a manipulated form of K562 feeder cells is an alternative option to effectively engage NK cells and trigger expansion [115]. Several clinical trials for adult and pediatric patients with leukemia, brain tumors such as medulloblastoma and glioblastoma, sarcomas such as osteosarcoma, Ewing sarcoma, rhabdomyosarcoma, and neuroblastoma have been conducted using *ex vivo* expanded NK cells. Recently, one of us (DAL) extensively reviewed the many clinical trials in which these cells have been used [111]. The safety, efficacy, and tolerability of NK cells in these clinical trials have shown promising results. However, clinical trials employing NK cells for the treatment of solid tumors have been less successful, which may be due to the impact of the suppressive tumor microenvironment [116].

Cancer cells secrete immune-suppressive mediators such as transforming growth factor beta (TGFβ), indoleamine 2,3-dioxygenase (IDO), and IL-10 which inhibit NK cells through suppression of cytotoxicity-related genes and signaling pathways. Overcoming these inhibitory effects through genetic or non-genetic modifications may enhance NK cell efficacy in targeting pediatric cancers. To overcome the suppressive effects of TGFβ on NK cells, Foltz et al. have shown that TGFβ imprinting during NK cell activation and expansion decreases NK cell sensitivity to TGFβ suppression [117]. CRISPR modification of primary and expanded NK cells using electroporation of Cas9/RNP targeting *TGFBR2* also has proven to be an effective mechanism to blunt NK cell inhibition upon exposure to TGFβ [118]. Using the CRISPR modification approach, there is evidence showing that gene-modified primary NK cells have enhanced antitumor effects, including *SOCS3* and *CISH* knockout NK cells [119–121]. Generating NK cells with enhanced specificity and adaptive mechanisms to overcome suppressive signals tilts the scale to support better outcomes for adoptive immunotherapy for pediatric cancers. An emerging technology in targeted therapy involves engineered NK cells known as chimeric antigen receptor (CAR)-NK cells. Cord blood-derived CAR-NK cells manipulated to express IL-15 and another CAR-NK line designed to target CD19 have shown potent antitumor activity against B cell leukemia [122]. Poor efficacy of transgene delivery in primary NK cells has limited the production of primary CAR-NK cells. However, a combination of Cas9/RNP and AAV6 transduction has shown to be highly efficient for CRISPR-directed gene insertion into primary NK cells which potentially may be used for production of primary CAR-NK cells [119, 123]. Altogether, adoptive cellular therapy with expanded, gene-modified, or CAR-expressing NK cells for pediatric cancers may serve as an effective treatment option for pediatric cancer patients. Additionally, the beneficial effects of these engineered NK cells may be enhanced through combination therapies with other antibodies, inhibitors, immune modulators, cytokines, and checkpoint inhibitors to achieve robust antitumor activity [111].

### Cancer vaccines

As their name suggests, cancer vaccines are substances that are administered to a patient in hopes of promoting an antitumor immune response. Although these agents can be designed for prophylactic use (e.g., the human papillomavirus vaccine to prevent cervical cancer [124]), we will focus on their use here as immunotherapies for already established cancers. Advancements in vaccine technology have yielded several platforms from which cancer vaccines can be developed [125]. These include cellular-based vaccines containing autologous or allogenic irradiated cancer cells [126], peptide-based vaccines which use small cancer-related peptides to stimulate MHC class I-presenting immune cells [127], DNA and RNA vaccines which trigger nucleic acid sensors and activate dendritic cells [128], and viral vector-based vaccines which naturally stimulate robust innate and adaptive immune responses [129]. Regardless of what form they take, cancer vaccines present tumor-associated antigens complexed with MHC molecules to B and T lymphocytes, setting off a cascade of events that ultimately promote an antitumor immune response [125]. The most important component of cancer vaccine design is antigen selection, which ideally should be expressed specifically on cancer cells, be necessary for their survival, and likewise be highly immunogenic. While few of any antigens meet all these criteria, the situation is exacerbated in the pediatric space due to the paucity of mutations and neoantigens present in the majority of childhood cancers. Despite this added challenge, clinical trials utilizing cancer vaccines in pediatric malignancies are currently underway.

A phase I/II study sponsored by the Baylor College of Medicine in collaboration with Texas Children’s Hospital seeks to test the safety and efficacy of metronomic Cytoxan chemotherapy combined with an allogeneic cancer vaccine in relapsed/refractory neuroblastoma (NCT01192555). Previous studies have shown that pediatric patients treated with cancer vaccines derived from neuroblastoma cells engineered to express interleukin-2, and the chemokine lymphotactin (XCL1) showed increased levels of tumor infiltration by T cells, eosinophils, dendritic cells, and NK cells [130]. Of the 28 patients treated with these vaccines in an early trial, 4 exhibited complete responses (including 2 sustained for ≥4 years), 2 had partial responses, and 5 had stable disease. The vaccine was also well tolerated, with severe adverse events being limited to 5 incidences of reversible panniculitis and 1 incidence of bone pain. The current study, which was initiated in 2010, reached its primary completion date in 2012 and is expected to be completed in 2026 [91].

Another phase I clinical trial at the University of Florida is currently recruiting pediatric patients with high-grade glioma to evaluate cancer vaccines comprised of dendritic cells “pre-loaded” with allogenic tumor RNA given in combination with GM-CSF (NCT03334305). This vaccine is part one of a two-step strategy which also entails a later infusion of tumor-specific T cells to support antitumor immunity. This study was initiated in 2017 with the primary purpose of determining the safety of this vaccination strategy, but no preliminary results were available at the time of this writing. Secondary outcomes include assessments of feasibility and antitumor immune responses, with assessments of progression-free and overall survival up to 8 years following treatment. The trial is expected to reach its primary completion date in 2022 and conclude 4 years later [90]. This site is also actively recruiting diffuse intrinsic pontine glioma (DIPG) patients for evaluation of a similar cancer vaccine intervention in combination with temozolomide chemotherapy, and is also expected to be completed in 2022 (NCT03396575) [92].

Investigators at Duke University have also recently extended investigation of their PEP-CMV cancer vaccine to pediatric medulloblastoma and malignant glioma patients (NCT03299309). Previous studies have shown that human cytomegalovirus (CMV) infection occurs in malignant gliomas, where it is implicated as a driver of important oncogenic pathways and glioma pathogenesis [131]. A 2006 phase I/II study showed that the administration of autologous CMV pp65 RNA-loaded dendritic cells was effective against adult glioblastoma, leading to significant increases in progression-free and overall survival. This led to the development of the “second generation” PEP-CMV vaccine, which as its names suggests, is a peptide vaccine derived from CMV antigens. While the oncogenic role of CMV infection in medulloblastoma is contested [132], the Duke phase I trial is currently seeking to enroll 30 patients, ages 3 to 35, for evaluation of this novel immunotherapy [99].

Studies at the University of California, San Francisco, are also underway with a phase I trial evaluating a peptide vaccine in children and young adults with DIPG and other gliomas (NCT02960230). This vaccine is based on the histone H3.3K27M mutation found in approximately 60% of high-grade pediatric glioma cases, which is known to drive tumorigenesis by silencing tumor suppressor genes [133, 134]. Patient accrual is expected to be completed shortly, and preliminary results are expected to be available in 2020 [100].

Two of the more clinically advanced trials are being conducted by the biotechnology company Gradalis, who is actively investigating the proprietary Vigil cancer vaccine for treatment of Ewing sarcoma (NCT02511132 and NCT03495921). Vigil immunotherapy is an autologous tumor cell product that is genetically engineered to express GM-CSF and shRNA for the protease furin, which normally activates TGFβ. Vigil has been evaluated extensively in advanced gynecological tumors (often in the context of other immunotherapies), producing encouraging results in regard to its safety and efficacy [135]. A multicenter phase IIb Vigil trial was initiated in 2015, in which pediatric Ewing sarcoma patients meeting the eligibility criteria receive either intradermal Vigil every 28 days for 4–12 administrations, or a combination of intravenous gemcitabine and docetaxel every 21 days. The primary objective of this trial is to determine the safety profile and overall survival of patients treated with Vigil *versus* chemotherapy. This study is expected to conclude in late 2019, but no preliminary results were available at the time of this writing [98]. A multicenter phase III trial is also underway as of 2018, which seeks to enroll 114 participants for treatment with Vigil and/or temozolomide plus irinotecan [96].

## Modulation of the tumor microenvironment

The tumor microenvironment (TME) is a major barrier to effective cancer treatment. In this section, we briefly discuss some of the measures that are being investigated to counteract the immunosuppressive milieu that characterizes the TME and how they are having an impact on the successful implementation of immunotherapy.

### Targeting macrophages

Macrophages are phagocytic cells of innate immunity known for their pro-inflammatory role against pathogens [136]. Tumor-associated macrophages (TAMs) are the chief regulators of the tumor microenvironment and can have either tumor-promoting or tumor-suppressing effects based on their functional states [136–138]. While the spatial and temporal dynamics of the equilibrium between these functional states are poorly understood, high TAM infiltration correlates with poor prognosis across a variety of cancer types [137]. TAMs are usually associated with pro-tumorigenic effects in the tumor microenvironment and promote immunosuppression through the release of cytokines and chemokines. Macrophage-derived IL-10, reactive oxygen species, or arginase can inhibit proliferation of lymphocytes in the microenvironment. Other macrophage-secreted factors, such as vascular endothelial growth factor (VEGF), platelet-derived growth factor, and matrix metallopeptidase-9 can induce angiogenesis in hypoxic areas of the tumor microenvironment. Macrophages can also promote metastasis through the CCL2/CSF-1 signaling axis [136].

Given their crucial role in tumorigenesis, two broad strategies have been employed to therapeutically target macrophages. One approach is to deplete the macrophage population in general, but this strategy is deemed non-ideal due to the role of macrophages in tissue homeostasis. Along these lines, ablating macrophage-derived factors or abolishing their effector functions have also been utilized. An alternate strategy involves polarizing the functional state to exhibit a tumor-suppressing phenotype. The effect of this phenotype is often pronounced through increased T cell infiltration which enhances antitumor immunity. Apart from these, key pathways responsible for infiltration, differentiation, and survival of macrophages in the microenvironment have also been targeted [137].

The rationale for targeting macrophages in childhood cancers is underscored by a number of recent studies in neuroblastoma. Pediatric neuroblastoma can have two different molecular phenotypes: tumors with amplified *MYCN* oncogene, deemed to be of high-risk clinically, or tumors without *MYCN* amplification which have a relatively better prognosis [139]. In neuroblastoma tumors without *MYCN* amplification, TAMs were shown to activate the STAT3 pathway in an IL-6 independent manner, leading to enhanced tumorigenesis. The presence of TAMs also led to the upregulated expression of the *MYCC* oncogene in neuroblastoma cells grown in culture [140]. In high-risk neuroblastoma, macrophages were found to suppress the efficacy of the anti-GD2 antibody dinutuximab. Depleting macrophages along with endothelial cells and mesenchymal stromal cells using the anti-CD105 antibody, TRC105, enhanced the efficacy of dinutuximab and increased overall survival in immunodeficient mice models when combined with adoptive transfer of activated NK cells [141].

Preclinical work on targeting macrophages has shown promise in animal models of cancer common in children. One approach used for this purpose is downregulating the M-CSF/CSF-1R axis, a pathway critical for proliferation and survival of macrophages, using CSF1-R inhibition. Upregulation of this pathway is frequently observed in cancer and is associated with a poor prognosis. CSF-1R blockade showed reduced tumor growth and enhanced survival either alone as a single agent or in combination with chemotherapy in immunocompetent models and correlated with enhanced T cell infiltration in various cancer types [142, 143]. In neuroblastoma, CSF1-R inhibition exhibited marginal benefit as a monotherapy in immunodeficient mice models but synergized with chemotherapy in combination. These results in models devoid of mature T cells show that targeting macrophages could be equally important even when T cell-mediated antitumor immunity is not prominent [144]. CSF-1R blockade has also been shown to synergize with PD-1/PD-L1 checkpoint inhibition and increase antitumor efficacy in a spontaneous model of high-risk neuroblastoma [145]. Eissler et al. further explored the mechanisms behind this synergy [146]. Anti-PD1 antibody polarized myeloid cells *in vitro* to a suppressive phenotype through M-CSF production from activated T cells. These suppressive myeloid cells in turn inhibited T cell proliferation by aiding the production of adenosine through upregulation of adenosine catabolizing enzymes and enhanced PD-L1 expression [146]. Antitumor efficacy was further increased by concurrent inhibition of CSF-1R and PD-1, which was associated with enhanced T cell infiltration through myeloid cell-derived chemokines CXCL9, CXCL10, and CXCL11. Similarly, macrophage reduction also enhanced antitumor efficacy following oncolytic virotherapy in xenograft models of Ewing sarcoma [147]. Both macrophages and stroma had an increase in antitumorigenic and decrease in pro-tumorigenic gene expression, along with a shift in the phenotype of TAMs to a more inflammatory state [147]. Recently, the plasminogen activator inhibitor (PAI-1) has been shown to promote macrophage infiltration and polarization towards a pro-tumorigenic “M2” phenotype through the p38MAPK/NF-κB/IL-6 axis [148]. PAI-1 expression is positively correlated with the presence of M2 macrophages in neuroblastoma and glioblastoma [148]. Thus PAI-1 could be a possible target for macrophage modulation in cancer. As macrophage recruitment and polarization are mediated by distinct domains of PAI-1, it could potentially be exploited in pediatric cancers to polarize macrophages to an antitumor phenotype without altering their infiltration [148].

Although growing evidence stresses the importance of macrophage modulation in immunotherapy, macrophage dependence could vary across tumor types. Even between two different xenograft models of Ewing sarcoma tested, only one was found to benefit from macrophage modulation while the other model showed marginal effect of the combination on antitumor efficacy compared with virotherapy alone [147]. Along these lines, in a recent characterization of the tumor microenvironment of diffuse intrinsic pontine glioma (DIPG), there was not a significant macrophage content in analyzed tumors [149].

There are a number of clinical trials targeting TAMs in cancers of adult patients at present (reviewed in [136]) which will likely open the doors for pediatric clinical testing in the future. Macrophage targeting is emerging as an important determinant of the success of immunotherapy in the pediatric setting. The challenge will be to characterize the tumor microenvironment in pediatric tumors and identify the dependence of a particular immunotherapeutic modality on macrophages.

### Targeting blood vessels

Abnormal vasculature is one of the defining features of cancer. The growing demand for nutrients and oxygen, coupled with rapid growth, leads to the formation of structurally and functionally impaired blood vessels in the tumor microenvironment. Leaky blood vessels and defective lymphatic drainage decrease perfusion, aggravate hypoxia, and lower the pH [150]. Collectively, these factors can alter antitumor immunity directly or indirectly. The direct effect is mediated through decreased immune cell infiltration and impaired effector function of these cells. Increased expression of VEGF during angiogenesis affects cell adhesion receptors of endothelial cells: integrin ligand receptor intercellular adhesion molecule 1 (ICAM-1) and vascular cell adhesion molecule 1 (VCAM-1), thus altering the infiltration of immune cells in the microenvironment [151]. Hypoxia and low pH directly impair the effector function of immune cells, and indirect effects, such as the release of cytokines and chemokines that recruit other immune cell subtypes, help create an immunosuppressive milieu [152, 153].

Two strategies commonly utilized to target VEGF signaling, anti-VEGF antibody, and small-molecule inhibitors of VEGF receptor tyrosine kinase, have been tested extensively in preclinical pediatric cancer models (reviewed in [154]). We mainly focus on the use of antiangiogenic agents to enhance immunotherapy in this section. The molecular hallmarks of angiogenesis are comparable in adult and pediatric tumors [154]. VEGF is a key mediator of angiogenesis in both cases. In clinical studies with adult patients, antiangiogenic therapies are found to synergize with cancer immunotherapy. A clinical trial investigating the combination of bevacizumab, a humanized monoclonal antibody against human VEGF-A, and the anti-CTLA-4 antibody ipilimumab in metastatic melanoma showed increased infiltration of CD8^+^ T cells and CD163^+^ dendritic macrophages [155]. The combination was also found to be well tolerated in glioblastoma [156]. These studies have prompted the testing of the antiangiogenic agents combined with immunotherapy in preclinical models of pediatric cancer. In an orthotopic xenograft model of human neuroblastoma, bevacizumab administered concurrently with GD2 CAR-T cells enhanced the infiltration of these cells in the tumor microenvironment compared with the T cell therapy alone. The synergistic effect on survival was observed even at low doses of bevacizumab, which alone exhibited marginal effects [157]. In murine melanoma models, the VEGF tyrosine kinase inhibitor sunitinib enhanced the antitumor response of an agnostic CD40-antibody. This finding was attributed to increased activation of dendritic cells and subsequent infiltration of cytotoxic T cells, along with reduced recruitment of myeloid-derived suppressor cells. The increased infiltration of T cells was associated with upregulation of ICAM-1 and VCAM-1 on endothelial cells [158].

Although these results are promising, there is still a dearth of clinical work in the pediatric population with the objective of decreasing angiogenesis to improve immunotherapy in an adjuvant setting. There are two clinical trials in pediatric osteosarcoma that combine antiangiogenic agents with immune checkpoint blockade at present: a phase I/II trial combines tyrosine kinase inhibitor famitinib with camrelizumab (NCT04044378) and a phase II trial combining apatinib with camrelizumab (NCT03359018) [89, 159].

An emerging strategy that could shape the use of antiangiogenic therapy in light of their role in enhancing the outcome of immunotherapy is the “vascular normalization” approach, based on evidence that some cytotoxic therapies have an enhanced antitumor effect when administered concurrently with antiangiogenic agents. This was deemed paradoxical as antiangiogenic agents deplete vessels supplying drugs to the tumor. This conundrum ostensibly led Jain et al. to propose the normalization hypothesis, where they posited that antiangiogenic therapy induces a transient state of normalization [151]. The use of antiangiogenic agents at a low dosing regimen to normalize blood vessels could significantly enhance the outcome of cytotoxic cancer therapies. This concept has since been validated independently through a number of preclinical and clinical studies. Along these lines, increased infiltration of GD2-CAR T cells in a preclinical model of neuroblastoma in an adjuvant setting with bevacizumab exhibited no significant decrease in micro-vessel number in tumors treated with a low dose antiangiogenic agent although synergy was clearly demonstrated with respect to survival benefit [157]. Sparse data in preclinical childhood cancer models cloud any judgment of the potential of the normalization approach in pediatric immunotherapy at present. Although there are significant challenges (including elucidating the factors that modulate the kinetics of vessel normalization, enhancing the window of normalization, and targeting alternate pathways during resistance), preclinical and clinical studies are underway to utilize this approach to enhance immunotherapy [151].

### Targeting myeloid-derived suppressor cells

Myeloid-derived suppressor cells (MDSCs) are a heterogeneous population of undifferentiated myeloid cells characterized by their ability to negatively regulate immune function, and thus aid tumor progression [160, 161]. The suppression of immune function by MDSCs is mediated by their production of arginase I and prostaglandin E2, reactive oxygen species (ROS), and release of immune suppressive cytokines [162–164]. There are two main sub-groups of MDSCs, polymorphonuclear or granular MDSCs (PMN-MDSCs) and monocytic MDSCs (M-MDSCs), and although both can impede antitumor immunity, M-MDSCs are generally thought to be more suppressive [165, 166].

A number of therapeutic strategies have been tested to target MDSCs. These approaches either interfere with their infiltration and activity or deplete them from the microenvironment (for a detailed review, refer to [167]). One approach exploits the upregulation of TNF-related apoptosis-induced ligand receptor 2 (TRAIL-R2) mediated apoptosis during endoplasmic reticulum (ER) stress in MDSCs to decrease its survival. An agonistic antibody of TRAIL-R2 (DS-8273a) was utilized to mimic this stress response pathway in advanced melanoma in a phase I trial. Although there were no objective responses, the number of MDSCs decreased in peripheral blood after the treatment. Three out of 6 patients also showed a decrease in intratumoral MDSCs [168].

Another approach to enhance antitumor immunity through terminally differentiated MDSCs has been utilized in advanced melanoma. The vitamin A derivative all-trans retinoic acid (ATRA), which suppresses retinoic acid signal transduction, was used for this purpose in an adjuvant setting with ipilimumab (NCT02403778) [169]. The treatment increased the frequency of MDSCs and decreased the frequency of mature myeloid cells in the peripheral blood. Some of these strategies have been extended to preclinical and clinical pediatric tumors. In pediatric xenograft models of osteosarcoma, ATRA was found to enhance the antitumor efficacy of GD2 CAR-T cells. A significant decrease in the number of monocytic MDSCs was observed while the remaining MDSCs had reduced suppressive activity on T cells [170]. Recently, immunotoxins have also been utilized to selectively deplete MDSCs in pediatric patient samples *in vitro*. An anti-CD33 antibody-calicheamicin toxin conjugate, gemtuzumab ozogamicin, was tested in neuroblastoma, Wilms tumor, Ewing sarcoma, rhabdomyosarcoma, and non-Hodgkin lymphoma. Depletion of MDSCs restored T cell proliferation and enhanced antitumor cytotoxicity of CAR-T cells in their respective functional assays [171]. The role of MDSCs in the success of other immunotherapies has also been tested in adult patients but this is yet to be tested in pediatric cancers. The clinical benefit of anti-CTLA4 antibody ipilimumab was found to depend upon the frequency of MDSCs [172]. Only the patients who responded to the checkpoint blockade had a lower frequency of M-MDSCs [172]. Although targeting MDSCs alone might not be sufficient, the field of pediatric oncology may greatly benefit from their modulation in an adjuvant setting.

### Targeting cancer-associated fibroblasts

Cancer-associated fibroblasts (CAFs) are a heterogeneous population of fibroblast-like cells largely associated with their supporting role in tumor progression through their release of cytokines and growth factors [173, 174]. The role of CAFs in pediatric tumor microenvironments has not been extensively characterized. In neuroblastoma, cancer-associated fibroblasts are the predominant source of prostaglandin E2 (PGE2) which promotes immunosuppression. In rhabdomyosarcoma, cancer cells have been shown to induce migration and invasion of fibroblasts and promote their progression through microRNA-loaded exosome cargo *in vitro* [175]. A recent pan-cancer analysis of The Cancer Genome Atlas data showed that extracellular matrix genes dysregulated in cancer correlated with activation of TGFβ signaling in CAFs. The dysregulation signature observed was linked to PD-1 blockade failure in the analysis [176].

Abolishing cancer-associated fibroblasts alone might not be ideal as an immunotherapy since their loss induced immunosuppression in preclinical pancreatic cancer models [177]. This strategy, however, was found to synergize with anti-CTLA-4 therapy in an adjuvant setting through its unleashing of checkpoint blockade targets during immune suppression. Although preclinical work on therapeutic targeting of CAFs in pediatric cancer models is limited, the results so far show that targeting CAFs could be a promising approach. Targeting CAF mediated production of PGE2 in neuroblastoma through inhibition of a key enzyme in its synthesis, microsomal prostaglandin E synthase-1, suppressed CAF migration and infiltration leading to reduced tumor growth and decreased angiogenesis [178]. In neuroblastoma, CAFs were also explored as an alternative to cell-based therapy utilizing autologous tumor cells to express pro-inflammatory cytokines. For this purpose, fibroblasts were modified genetically to co-express IL-2 and IL-12 in a syngeneic mouse model. The co-injection of these genetically engineered fibroblasts with tumor cells completely inhibited tumor induction. The mice models of neuroblastoma were even resistant to tumor progression upon re-challenge at a different site after 3 months, thus exhibiting systemic immunological memory [179]. Further work is necessary to characterize the importance for CAFs in pediatric immunotherapy.

## Future perspectives: challenges and opportunities

The landscape for cancer therapy has seen a dramatic shift over the last decade as immunotherapeutic interventions continue to mature and increasingly find their way from bench to bedside. Although there have been several laudable successes, the field is still clearly in its infancy and there is considerable room for future improvements. This prospect is doubly true for pediatric cancers which lag behind advancements seen in adult malignancies because of the unique set of circumstances and challenges inherent to these diseases. Despite some commonalities, pediatric tumors are fundamentally different than their adult counterparts. Most pediatric cancers arise from embryonal cells as opposed to epithelial cells, for example, and are likewise thought to result from transcriptional abnormalities, copy number variants, and chromosomal rearrangements rather than an accumulation of nonsynonymous genetic mutations [180]. Consequently, one of the defining traits of pediatric tumors is their low mutational burden and relative lack of neoantigen expression, which limits their susceptibility to immune targeting [65]. This limitation is evident in the number and nature of FDA-approved immunotherapies discussed in this review, as the most successful therapies available to date essentially still target normal cell surface proteins like CD19. The immune response itself can also be markedly different in adults and children, and lessons learned from the use of immunotherapy in the former may not be wholly applicable to the latter [181]. Another lingering concern is toxicity. While immunotherapies are generally thought to exhibit fewer long-term toxicities than chemotherapy and radiation, short-term adverse events are extremely common following the induction of these therapies and vary in severity depending on the agent and its intended target. These can range from minor inconveniences like fevers, headaches, and chills, to more serious events like myalgia, autoimmunity, neurotoxicity, and opportunistic infections; some of these events (e.g*.*, cytokine release syndrome) can potentially be life-threatening. The immunosuppressive tumor microenvironment also remains an ever-present obstacle for solid cancers, as it can diminish or nullify any benefit that might come from immunotherapeutic intervention.

While there is likely no “magic bullet” solution to these issues on the horizon, combining multiple immunotherapies is one strategy that may help mitigate some of the challenges facing pediatric cancer immunotherapy. For example, checkpoint inhibitors like pembrolizumab and ipilimumab bolster T cell activity through distinct mechanisms—PD-1 inhibition increases cytotoxic T lymphocyte proliferation, whereas CTLA-4 inhibition helps these cells remain activated. A phase I adult metastatic melanoma trial showed that concurrent PD-1/CTLA-4 signaling blockade resulted in improved antitumor efficacy, with 53% of patients showing an objective response with tumor reductions ≥ 80%, all while maintaining an acceptable safety profile [182, 183]. Phase I/II studies testing the combined use of these therapies in pediatric patients with recurrent or refractory solid tumors are also underway, although data from these remain forthcoming [69–71]. Checkpoint inhibitors are increasingly finding their way as components of other combination therapies as well, thanks to their general safety and the importance of T cell activation in the establishment of antitumor immunity [184, 185]. This concept includes most if not all of the modalities summarized in this review, including CAR-T and adoptive cell-based therapies [186, 187], monoclonal and bispecific antibodies [188, 189], and oncolytic viruses [190–192]. The rationale for combining these therapies extends beyond simply improving antitumor efficacy, as careful selection of agents with non-overlapping toxicities may also help mitigate the incidence and/or severity of treatment-related adverse effects.

While it is inarguable that there are still significant challenges that need to be fully addressed, the future of immunotherapy in pediatric cancers should be viewed with optimism. The last decade alone has seen five distinct immunotherapies obtain FDA approval for various pediatric malignancies, and several upcoming modalities have the potential to join their ranks in the coming years. New therapies will also inevitably come into development as our understanding of these diseases, the complexities of tumor microenvironment, and the intricacies of the pediatric immune system come into sharper focus. Leveraging the strengths of these therapies, particularly through rational drug combinations, will hopefully allow us to better care for pediatric cancer patients both by increasing their survival and improving their overall quality of life.
